# Pathways among the nursing practice environment, job burnout, and job satisfaction to intention to leave: a cross-sectional study conducted in Taiwan

**DOI:** 10.1590/1980-220X-REEUSP-2024-0025en

**Published:** 2024-10-11

**Authors:** Shu-Fen Wu, Ching-Yun Ching, Hsiu-Chen Liao, Ruey-Hsia Wang

**Affiliations:** 1 Kaohsiung Medical University, College of Nursing, Kaohsiung, Taiwan.; 2 Yuh-Ing Junior College of Health Care & Management, Department of Nursing, Kaohsiung, Taiwan.; 3 Yuan’s General Hospital, Department of Nursing, Kaohsiung, Taiwan.; 4 Kaohsiung Medical University Hospital, Department of Medical Research, Kaohsiung, Taiwan.

**Keywords:** Environment, Burnout, Psychological, Job Satisfaction, Personnel Turnover, Meio Ambiente, Esgotamento Psicológico, Satisfação no Emprego, Reorganização de Recursos Humanos, Ambiente, Agotamiento Psicológico, Satisfacción en el Trabajo, Reorganización del Personal

## Abstract

**Objective:**

The purpose of this study was to explore the pathways among the practice environment, job burnout, and job satisfaction and their relationships with the intention to leave among clinical nurses.

**Method:**

A cross-sectional survey was administered to 310 nursing staff members working in regional teaching hospitals in southern Taiwan. The instruments used included the practice environment, job satisfaction, job burnout, and sociodemographic characteristics questionnaires.

**Results:**

Structural equation modeling indicated that job satisfaction (β = –0.167) and job burnout (β = 0.361) were significantly and directly associated with the intention to leave, whereas the practice environment was significantly and directly associated with job satisfaction (β = 0.447). The practice environment (β_indirect_ = –0.075) and job burnout (β_indirect_ = 0.060) were significantly and indirectly associated with the intention to leave through job satisfaction.

**Conclusion:**

Job burnout and job satisfaction are directly associated with the intention to leave. Therefore, improving the practice environment and subsequent job burnout could be strategies to improve job satisfaction and decrease the intention to leave.

## RESUMO

**Objetivo:**

O objetivo deste estudo foi explorar os caminhos entre o ambiente de prática, o esgotamento profissional e a satisfação no trabalho e suas relações com a intenção de sair entre enfermeiros clínicos.

**Método:**

Foi realizado um estudo transversal com 310 funcionários de enfermagem que trabalham em hospitais universitários regionais no sul de Taiwan. Os instrumentos utilizados incluíram questionários de ambiente de prática, satisfação no trabalho, esgotamento profissional e características sociodemográficas.

**Resultados:**

A modelagem de equações estruturais indicou que a satisfação no trabalho (β = –0,167) e o esgotamento profissional (β = 0,361) estavam significativa e diretamente associados à intenção de sair, enquanto o ambiente de prática estava significativa e diretamente associado à satisfação no trabalho (β = 0,447). O ambiente de prática (βindireto = –0,075) e o esgotamento profissional (βindireto = 0,060) estiveram significativa e indiretamente associados à intenção de sair por meio da satisfação no trabalho.

**Conclusão:**

O esgotamento profissional e a satisfação profissional estão diretamente associados à intenção de sair. Portanto, a melhoria do ambiente de prática e o subsequente esgotamento profissional poderiam ser estratégias para melhorar a satisfação no trabalho e diminuir a intenção de sair.

## RESUMEN

**Objetivo:**

Para explorar la relación entre el entorno de práctica de las enfermeras clínicas, el agotamiento del trabajo, la satisfacción laboral y su relación con la tendencia a dejar el trabajo.

**Métodos:**

Se realizó una encuesta transversal de 310 enfermeros en hospitales de enseñanza en el sur de Taiwán. Las herramientas utilizadas incluyeron el entorno de la práctica, la satisfacción laboral, el agotamiento laboral y el cuestionario de características sociodemográficas.

**Resultados:**

El modelo de ecuaciones estructurales mostró que la satisfacción en el trabajo (β = –0.167) y el agotamiento en el trabajo (β = 0.361) se correlacionaron significativamente directamente con la tendencia a la separación del trabajo, mientras que el entorno de la pasantía se correlacionó significativamente directamente con la satisfacción en el trabajo (β = 0.447). El entorno de práctica (β-indirect = –0.075) y el agotamiento en el trabajo (β-indirect = 0.060) se correlacionaron significativamente e indirectamente con la intención de separación por satisfacción laboral.

**Conclusión:**

El agotamiento del trabajo y la satisfacción laboral están directamente relacionados con la tendencia a abandonar el trabajo. Por lo tanto, mejorar el entorno de pasantías y el agotamiento laboral resultante puede ser una estrategia para aumentar la satisfacción laboral y disminuir la tendencia a dejar el trabajo.

## INTRODUCTION

The nursing workforce constitutes half of the global healthcare system^(1)^. However, the intention to leave among nursing staff is at a crisis level worldwide^(2,3)^. The intention to leave is an employee’s plan to leave his or her current institute and find an alternative job. Although the intention to leave does not necessarily mean actual turnover^(4)^, a higher intention to leave leads to a high turnover rate^(5)^. The literature has shown that nurses’ intention to leave poses a severe threat to the quality of care and patient outcomes^(6)^. As noted in previous studies, the intention to leave may impact healthcare service stability and performance and increase healthcare system costs^(4,6)^. The evidence shows that nurses have a high prevalence of intentions to leave, with 26.0% in Saudi Arabia, 36% in the United States, 18.8%–41.4% in African countries, and 26%–40% in China^(5,7,8)^. Understanding pathways to the intention to leave will be beneficial for designing interventions to reduce the intention to leave.

Many factors might contribute to a high intention to leave. Recent research has identified organizational factors and individual factors that are associated with nurses’ intention to leave. The array of influencing factors includes sociodemographic variables, job burnout, job satisfaction and the practice environment^(6,9)^. Job satisfaction can be considered a fulfillment of desired needs within work settings, happiness or gratifying emotional responses toward working conditions, and job value or equity^(10)^. Job satisfaction was negatively related to job burnout and the intention to leave. Previous cross-sectional studies reported that nurses with greater job satisfaction reported lower intentions to leave^(11,12)^. Additionally, structural equation modeling (SEM) also suggested that greater job satisfaction could directly or indirectly affect the lower intention to leave^(5,13)^. Therefore, greater job satisfaction might be directly associated with lower intentions to leave.

Job burnout is defined as a state of physical, mental, emotional, and social exhaustion resulting from the negative effects of unmanaged occupational stress and inadequate managerial and social support, which reduces motivation for work^(14)^. Previous studies have shown that a high level of job burnout is associated with greater intention to leave^(6,15)^. Studies using SEM have also shown that job burnout is directly associated with job satisfaction and the intention to leave^(5,16)^. Ran et al.^(5)^ reported that job burnout might be indirectly associated with the intention to leave through job satisfaction. Thus, high job burnout among nurses might not only directly lead to high intention to leave and low job satisfaction but also indirectly affect high intention to leave through low job satisfaction.

The practice environment is an organizational characteristic of the work setting that affects professional nursing practice^(17)^. The influence of the practice environment on job burnout, job satisfaction, and intention to leave has been extensively debated. Nurses who work in better practice environments have reported significantly lower levels of job burnout^(9,16)^. Extensive research has examined the relationship between the practice environment and job satisfaction. For example, a previous study demonstrated that the practice environment is directly associated with job satisfaction^(16)^. Nurses with better practice environments report greater job satisfaction^(11,18)^. Furthermore, as noted in previous studies, there is a significant correlation between the practice environment and the intention to leave^(11,16)^. A systematic review and meta-analysis revealed that better practice environments were significantly correlated with lower intentions to leave^(15)^. Another study revealed that poor practice environments indirectly increase nurses’ intention to leave by increasing job burnout^(8)^. Furthermore, studies using SEM have shown that the practice environment is directly linked to job satisfaction and is indirectly linked to the intention to leave through job satisfaction^(16,18)^. Accordingly, the practice environment may be directly associated with job burnout, job satisfaction and the intention to leave and indirectly associated with the intention to leave through job burnout and job satisfaction. Nevertheless, these hypothetical pathways have not been tested.

**Figure 1:**
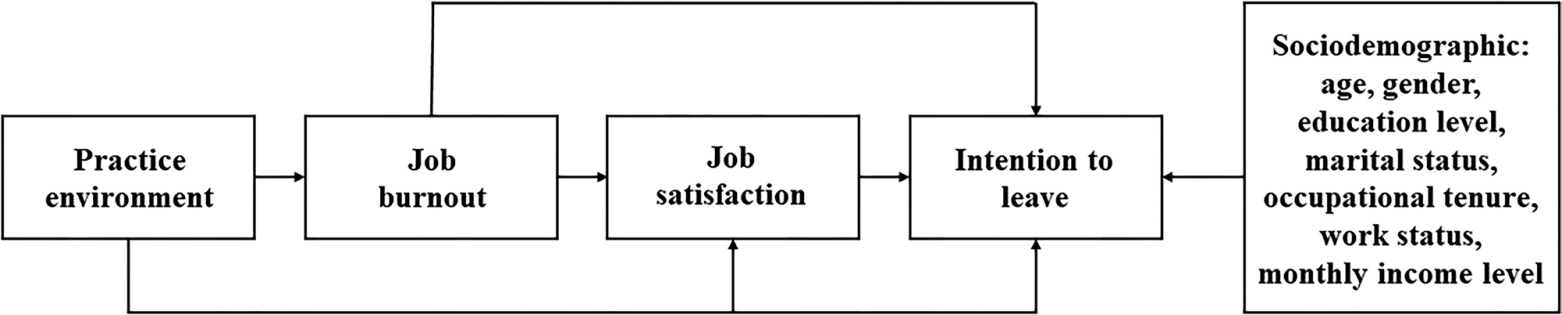
The hypothesized pathways among sociodemographic, practice environment, job burnout, and job satisfaction to intention to leave.

Sociodemographic characteristics, including age, gender, education level, marital status, occupational tenure, monthly income level, and working status, are associated with the intention to leave^(9,15)^. Previous studies have reported a high level of intention to leave among nurses who were younger^(9)^, single and had less than ten years of occupational tenure^(5,12)^. Nurses with a master’s degree reported lower intentions to leave than did nurses with a bachelor’s degree^(9,12)^. Conversely, in another study, nurses who had a master’s degree reported greater intentions to leave^(9,18)^. In a study by Ran et al.^(5)^, education level and monthly income level were directly associated with the intention to leave. Therefore, sociodemographic characteristics should not be neglected when constructing pathways to the intention to leave. As we know, no existing theoretical framework has described the pathways among the nursing practice environment, job burnout, and job satisfaction and the intention to leave. We hypothesized a model according to previous literature (Figure 1). The purpose of this study was to test the hypothesized model.

## METHOD

### Design of the Study

This was a cross-sectional observational study.

### Location, Population and Sample Definition

This study was conducted at a private general hospital in southern Taiwan. The study enrolled registered nurses via convenience sampling. The required sample size was estimated via structural equation modeling^(19)^. Given the anticipated medium effect size of 0.3, power level of 0.80, and α level of 0.05, a minimum sample size of 150 was needed. Data were collected from August to October 2018, and 332 self-report questionnaires were distributed in wards other than the intensive care unit. Ultimately, 310 of the 332 registered nurses completed the questionnaires, for a response rate of 93.37%.

### Selection Criteria

In this study, registered nurses who had worked for more than 3 months were recruited. Registered nurses who were pregnant or breastfeeding at the time of data collection were excluded because these characteristics come with physical, emotional, and related work stress challenges that impact women’s experience in the workplace, job satisfaction, burnout level and intention to leave^(20)^.

### Data Collection

Before data collection, the consent of the directors of the nursing wards was obtained. For the selection of eligible nurses, all of the lists indicated were selected by a researcher in advance and according to the sampling criteria through the human resources system. Three trained researcher assistants distributed sealed envelopes, including an information sheet, consent form, and self-report questionnaire, to eligible nurses in the nursing wards. After the eligible nurses completed the ­self-report questionnaire, they put the questionnaires into a box. The researchers subsequently collected questionnaires from the box and conducted the data analysis. The self-report questionnaire included the following parts.

### Practice Environment

The 31-item Chinese version of the Practice Environment Scale of the Nursing Work Index (C-PES-NWI), which was translated from the English version, was used to measure nursing practice environments^(21)^. Each item was rated on a 4-point Likert scale ranging from strongly disagree (1 point) to strongly agree (4 points). A higher score reflected nurses’ agreement that the practice environment was favorable^(17)^. The Chinese version of the C-PES-NWI scale has excellent reliability and validity. The Cronbach’s alpha for the subscale ranges from 0.69 to 0.88^(21)^. In the present study, the Cronbach’s α for the overall scale was 0.93.

### Job Satisfaction

The 12-item Chinese version of the Occupational Stress Indicator 2 (C-OSI-2) - Job Satisfaction Survey translated from the English version^(22)^ was used to measure the participants’ job satisfaction. Each item was rated on a 6-point Likert scale ranging from strongly dissatisfied (1 point) to strongly satisfied (6 points). A higher score indicates greater job satisfaction. The Cronbach’s alpha for the C-OSI-2-Job Satisfaction scale was 0.93^(22)^. In the present study, the Cronbach’s α for the overall scale was 0.94.

### Job Burnout

The 22-item Chinese version of the Maslach Burnout Inventory Human Services Survey (MBI-HSS) was used to measure the job burnout of participants^(23)^. The MBI-HSS has been widely used for measuring occupational burnout among healthcare workers; it has 22 statements and is categorized into three dimensions: emotional exhaustion (9 items), depersonalization (5 items), and personal accomplishment (8 items)^(24)^. Each item is rated on a 7-point Likert scale ranging from never experiencing such a feeling (0 points) to experiencing such feelings every day (6 points). Personal accomplishment was reverse scored. A higher total score represented more serious burnout. The Chinese version of the MBI-HSS scale has been shown to have excellent reliability and validity. The Cronbach’s α for the subscale ranges from 0.68 to 0.87^(22,25)^. In the present study, the Cronbach’s α for the overall scale was 0.91.

### Intention to Leave

Previous studies have used one question to assess the intention to leave^(13)^. This study also used a single question, “How frequently do you think about leaving your job within the next year?”, to assess the participants’ intention to leave on a ­five-point scale ranging from never (1 point) to always (5 points). A higher score indicated that the participants had a greater intention to leave.

### Sociodemographic Characteristics

Information on age, gender, education level, marital status, occupational tenure, work status (day shift or shift rotation), and monthly income was collected.

### Data Analysis

The statistical analyses were performed via IBM SPSS statistics software (version 25.0). Descriptive statistics were used to describe the distributions of the variables in this study. T tests, one-way ANOVAs and Pearson’s correlation were used to examine the associations between variables. SEM was conducted with AMOS statistics software (version 24.0) and used to examine the hypothesized model. The model was considered to have an acceptable fit if the ratio of chi-square/df < 5, goodness-of-fit index (GFI), normal-fit index (NFI), comparative fit index (CFI), and incremental fit index (IFI) were greater than 0.90 and if the root mean square error of approximation (RMSEA) ≤ 0.10 and standardized root mean square residual (SRMR) were lower than 0.08^(26)^. The bias-corrected percentile bootstrap test was used to examine the direct, indirect, and total effects of the hypothesized pathways. A β coefficient was considered significant when the 95% confidence intervals (CIs) did not include 0. If the model did not fit the data well, the model was further modified on the basis of the modification index.

### Ethical Aspects

The study was reviewed by the hospital’s institutional review board (No. 20170504B) to ensure full protection of the rights of the study participants. In addition, the participants were informed of the study’s aim and data collection process. All participants provided informed consent and completed the questionnaire. All the data were treated confidentially and anonymously.

## RESULTS

### Distribution of Sociodemographic Characteristics, Practice Environment, Job Burnout, Job Satisfaction, and Intention to Leave and Their Relationships

The distributions of sociodemographic characteristics and the intention to leave are presented in Table 1. The mean age of the participants was 34.88 years (*SD* = 9.68), the mean occupational tenure was 9.89 years (*SD* = 9.18), and approximately 71.94% worked rotating shifts. Approximately 36.77% (*n* = 114) of the participants often or always expressed the intention to leave their job within a year. As shown in Table 1, the intention to leave was significantly greater for nurses who were younger than 30 years, those who were single, those with an occupational tenure of less than 10 years, and those who worked rotating shifts.

**Table 1:** Distribution of sociodemographic characteristics by intention to leave – Taiwan, Republic of China, 2018.

Variable	n (%) or Mean + SD	Intention to leave Mean ± SD	t/F	p value
**Age group (year)**				
a. < 30	128 (41.29)	3.48 ± 1.03	10.863	< .001
b. 31–40	102 (32.90)	3.14 ± 1.05		
c. 41–50	56 (18.07)	2.71 ± 1.11		
d. > 50	24 (7.74)	2.42 ± 1.25		
Post hoc (Scheffe’s)		a > c, d
**Gender**				
Male	5 (1.61)	2.20 ± 1.10	–1.913	.057
Female	305 (98.39)	3.16 ± 1.11		
**Education level**		
Senior high school	85 (27.42)	2.99 ± 1.27	–1.522	.129
Above university	225 (72.58)	3.20 ± 1.05		
**Marital status**				
a. Single	155 (50.00)	3.39 ± 1.02	8.099	< .001
b. Married/partnered	148 (47.74)	2.91 ± 1.14		
c. Other	7 (2.26)	2.71 ± 1.50		
Post hoc (Scheffe’s)		a > b
**Occupational tenure (year)**			
a. < 3 years	90 (29.03)	3.41 ± 1.20	8.861	< .001
b. 3.01-10 years	116 (37.42)	3.26 ± 0.89		
c. > 10 years	104 (33.55)	2.79 ± 1.20		
Post hoc (Scheffe’s)		a, b > c	
**Working status**				
Day shift	87 (28.06)	2.64 ± 1.14	–5.129	< .001
Shift rotation	223 (71.94)	3.34 ± 1.05		
**Monthly income level**				
a. < 35,000 NTD$	80 (25.81)	3.13 ± 1.18	2.507	.083
b. 35,001–45,000 NTD$	182 (58.71)	3.24 ± 1.08		
c. > 45,000 NTD$	48 (15.48)	2.83 ± 1.12		
**Intention to leave**				
Never	27 (8.71)			
Seldom	54 (17.42)			
Sometimes	115 (37.10)			
Often	75 (24.19)			
Always	39 (12.58)			

Legend: n = number of respondents.

As shown in Table 2, the average total scores for the practice environment, job burnout, job satisfaction, and intention to leave were 87.95 (*SD* = 7.57), 58.13 (*SD* = 19.54), 45.34 (*SD* = 6.63), and 3.15 (*SD* = 1.12), respectively. The correlation between job burnout and intention to leave was significant and positive. The results also revealed a significant and negative correlation between nurses’ job satisfaction and intention to leave. Furthermore, the practice environment was not significantly correlated with the intention to leave or job burnout. Finally, the results suggested that job satisfaction was significantly correlated with the practice environment and job burnout.

**Table 2:** Description and Pearson’s correlations of the practice environment, job burnout, job satisfaction, and intention to leave – Taiwan, Republic of China, 2018.

Variables	Mean (SD)	2	3	4
1. Practice environment	87.95 (7.57)	.007	.445^***^	–.057
2. Job burnout	58.31 (19.54)		–.354^**^	.458^***^
3. Job satisfaction	45.34 (6.63)			–.304^***^
4. Intention to leave	3.15 (1.12)			

Legend: n = number of respondents

*p < .05

**p < .01

***p < .001.

### Path Analysis for the Hypothesized Model

**Figure 2:**
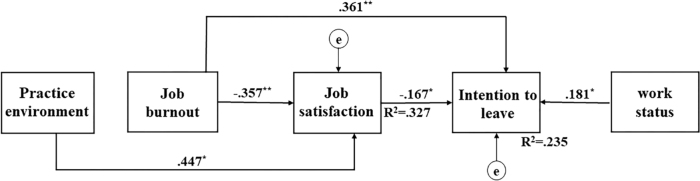
Direct pathway coefficients of sociodemographic, practice environment, job burnout, and job satisfaction and their relationships with intention to leave.

Note. *p values < .05 indicate significant results; **p values < .01 indicate significant results; ***p values < .001 indicate significant results.

The paths from gender, education level, and monthly income to the intention to leave and the pathways from the practice environment to job burnout and the intention to leave were not tested in the first path analysis because no significant associations were found in the bivariate correlation analysis. After the first SEM, the fit indices of the hypothesized model were χ^2^/df = 29.47, GFI = 0.59, NFI = 0.22, CFI = 0.22, IFI = 0.23, SRMR = 0.27, and RMSEA = 0.30. The hypothesized model did not fit the data well and required further modification. After SEM was conducted two times, the fit indices of the final model were χ^2^/df = 4.17, GFI = 0.98, NFI = 0.91, CFI = 0.93, IFI = 0.93, SRMR = 0.07, and RMSEA = 0.10. As shown in Figure 2, the standardized path coefficients were all significant. Specifically, the practice environment was significantly and directly associated with job satisfaction (β = 0.447, 95% CI, 0.357–0.521, *p* = .010). Job burnout was significantly and directly associated with job satisfaction (β = –0.357, 95% CI, –0.451– –0.271, *p* = .009) and the intention to leave (β = 0.361, 95% CI, 0.273–0.464, *p* = .007). Job satisfaction was significantly and directly associated with the intention to leave (β = –0.167, 95% CI, –0.275– –0.065, *p* = .010). In terms of sociodemographic data, only working status was significantly and directly associated with the intention to leave (β = 0.181, 95% CI, 0.065–0.286, *p* = .010).

In terms of indirect effects, the practice environment (β_indirect_ = –0.075, 95% CI, –0.126– –0.026, *p* = .012) and job burnout (β_indirect_ = 0.060, 95% CI, 0.019–0.104, *p* = .009) were significantly and indirectly associated with the intention to leave through job satisfaction. In the model, 23.5% of the variance in intention to leave was explained by work status (β = 0.181), job satisfaction (β = –0.167), and, to a great extent, job burnout (β = 0.361). This model also explains the influence of job burnout, work status and job satisfaction on the intention to leave. In this model, job satisfaction explained 32.7% of the variance in the practice environment and job burnout (Figure 2).

## DISCUSSION

More than one-third (36.77%) of the nurses stated that they “often” or “always” had the intention to leave their job within a year. This finding echoes previous studies from other countries that reported that nurses’ intention to leave ranged from 28.4% to 40.0%^(5,13)^. Working status was the only sociodemographic variable that was significantly and directly associated with the intention to leave. In this study, the intention to leave was more common among nurses who worked shift rotations, which is in line with the findings of previous studies^(5,27)^. Shift rotation may lead to considerable stress and workload and may ultimately contribute to a greater intention to leave among nurses^(26)^. Therefore, nurse managers should be aware of the intention to leave among nurses who work rotating shifts.

An important finding of this study was that greater job burnout was significantly and directly associated with greater intention to leave. This finding was consistent with a previous study^(16)^. Additionally, the standardized path coefficient from job burnout to job satisfaction was negative, which is consistent with the findings of previous studies^(18,28)^. Job burnout was significantly and indirectly associated with the intention to leave through job satisfaction, which was consistent with the findings of previous studies^(5,28)^. A previous study confirmed that increasing job burnout increases the likelihood of low job satisfaction and the intention to leave^(5)^. In this study, according to the standardized β coefficient, job burnout was more directly associated with the intention to leave than job satisfaction was. This means that job burnout plays a crucial role in the association with the intention to leave. Reducing job burnout might have a more beneficial effect than increasing job satisfaction to reduce the intention to leave. Given that job burnout directly and indirectly affects the intention to leave, improving job burnout is essential for reducing nurses’ intention to leave. Systematic reviews and ­meta-analyses have indicated that self-care workshops, mindfulness and meditation training can be adopted to decrease nurses’ job burnout^(29)^. Furthermore, creating a positive, supportive, and collaborative work environment and reducing patient-to-nurse ratios could also reduce nurses’ job burnout^(8,18)^. Managers can consider applying these strategies to reduce job burnout among nurses as a means of reducing their intention to leave.

In this study, job satisfaction was directly associated with the intention to leave, which is consistent with the findings of a previous study^(5)^. Moreover, the current study revealed that job burnout and the practice environment could be indirectly associated with the intention to leave through job satisfaction, which is consistent with the findings of previous studies^(5,30)^. Improving job satisfaction is essential for reducing nurses’ intention to leave. Previous studies have shown that improving salaries, reducing workloads, and encouraging peer cohesion among nurses could improve job satisfaction and reduce the intention to leave^(12,28)^. Hence, nursing managers can plan strategies to improve job satisfaction and reduce nurses’ intention to leave.

A direct path from the practice environment to job burnout was hypothesized but was not supported in this study. This result is inconsistent with that of a previous study conducted with SEM^(31)^ but is similar to the findings of another study^(25)^. In the present study, the practice environment did not directly affect job burnout, which may be due to differences in the professional climate or culture in the practice environment. More in-depth studies are needed to confirm this hypothesis. In the present study, the practice environment was directly associated with job satisfaction, which is consistent with the findings of previous studies^(18,29)^. Additionally, the practice environment was not directly associated with the intention to leave but was indirectly associated with the intention to leave but was indirectly associated with the intention to leave though job satisfaction, which was consistent with the findings of previous studies^(8,29)^. Some strategies were supported, such as providing adequate staffing and resources and enhancing nurses’ participation in hospital affairs as ways to establish a favorable practice environment, which can improve job satisfaction and decrease the intention to leave. Nursing managers can adapt these strategies to improve nurses’ practice environments and job satisfaction and reduce their intention to leave.

This study has several limitations. First, it employed a cross-sectional design, which cannot establish causal relationships. Second, these findings were obtained from a private hospital in southern Taiwan and may not be generalizable to other types of hospitals or countries. In the future, research should include participants from other types of hospitals and countries. A longitudinal study design is necessary to establish causal relationships in the supported model. Finally, because only one question was used to collect data on the intention to leave, there may be insufficient evidence to comprehensively assess the participants’ intention to leave. We suggest that more comprehensive scales should be used in future research.

## CONCLUSION

This study provides evidence to explain the factors and pathways associated with the intention to leave. The findings of this study indicate that the practice environment, job satisfaction, job burnout, and working status should be considered simultaneously to reduce nurses’ intention to leave. Job burnout and job satisfaction are directly associated with the intention to leave, and the practice environment and job burnout are directly associated with job satisfaction. Improving the practice environment and decreasing job burnout could be strategies to improve job satisfaction and decrease the intention to leave. This association has been empirically demonstrated. Hence, managers should pay more attention to creating a favorable practice environment to reduce job burnout, improve job satisfaction and decrease the intention to leave among clinical nurses.
